# Kynu inhibition mitigates bile duct ischemic injury by rewiring tryptophan metabolism to restore tight junction integrity

**DOI:** 10.1186/s10020-025-01310-6

**Published:** 2025-08-19

**Authors:** Guoqing Bao, Siliang Zhang, Zhengchen Ye, Ding Luo, Pinduan Bi, Bin Yang

**Affiliations:** Department of Hepatobiliary Surgery, The First Affiliated Hospital of Kunming Medical University, No. 295, Xichang Road, Wuhua District, Kunming, China

**Keywords:** Bile duct barrier, Ischemic injury, Tight junction, Kynu, Tryptophan metabolism

## Abstract

**Background:**

Disruption in bile duct barrier function contributes to hepatocyte toxicity in ischemia–reperfusion injury, often leading to surgical complications in liver resection, transplantation, and hemorrhagic shock. However, the underlying mechanisms remain incompletely understood.

**Methods:**

Transcriptomic and proteomic analyses were conducted to examine tryptophan (Trp) metabolism in a Pringle maneuver-induced bile duct injury rat model; Hypoxia/Reoxygenation (H/R) was used to establish an in vitro cholangiocyte injury model. Cholangiocyte injury was assessed via hematoxylin and eosin (H&E) staining, Ki67/myeloperoxidase (MPO) immunohistochemistry, transmission electron microscopy (TEM), and TUNEL/CK19 co-staining. Tight junction integrity was evaluated by measuring transepithelial electrical resistance (TEER), inulin permeability, and confocal immunofluorescence (IF) for ZO-1/CK19 co-staining. Gene expression was quantified using RT-qPCR and Western blotting, while metabolites were analyzed via liquid chromatography-tandem mass spectrometry (LC–MS/MS).

**Results:**

Significant alterations in Trp metabolism-related genes (Kynu, Haao, Kat1/Kat2) and metabolites were observed. Continuous Pringle maneuver resulted in elevated levels of 3-hydroxyanthranilic acid (3-HAA) and quinolinic acid (QA), a decreased xanthurenic acid (XA) level. In vitro, Kynu inhibition, using shRNA or the inhibitor benserazide (BSZ), ameliorated tight junction impairment and attenuated inflammatory damage in hypoxic biliary epithelial cells. In vivo, post-ischemia Kynu blockade reduced bile duct damage, inflammation, and biliary barrier permeability. Proteome analysis revealed that Kynu inhibition decreased 3-HAA, AA and QA levels while increased XA level. Notably, XA (but not AA or QA) treatment restored cell junction integrity under hypoxic conditions and modulated cytokine expression, potentially via ZO1 regulation through the GluR2/CX50 pathway. By day 7, BSZ or XA administration reduced serum bilirubin levels and mitigated of bile duct hyperplasia.

**Conclusion:**

Our findings demonstrated that Kynu inhibition alleviates bile duct ischemic injury by reprogramming dysregulated tryptophan metabolism, particularly through XA upregulation. This modulation may restore tight junction function via the GluR2/3/CX50-ZO1 axis, thereby preserving blood-biliary barrier integrity. Targeting Kynu represents a promising therapeutic strategy for ischemia-induced bile duct injury.

**Supplementary Information:**

The online version contains supplementary material available at 10.1186/s10020-025-01310-6.

## Introduction

Bile ducts ischemic injury is associated with high morbidity and mortality in patients following liver trauma, liver resection, and transplantation (Chen et al. 2021). Biliary complications, often referred to as the “Achilles heel” of liver transplantation, r remain one of the most challenging issues despite advancements in surgical techniques and experience (de Vries et al. 2018). These complications negatively impact graft survival and are often difficult to prevent and treat (Fujiki et al. 2017). Unfortunately, despite its profound clinical significance, there are no effective treatments for bile ducts ischemic injury (Guan et al. 2021). Therefore, developing more efficient and clinically compatible strategies to combat bile duct ischemia will improve patient prognosis.

Our previously study and other research have identified that bile duct injury may be associated with increased permeability of bile duct barrier due to loss of tight junctions (TJs). Small ducts are lined with small cholangiocytes, while larger ducts are lined with larger columnar epithelial cells, which maintains bile flow (Luo et al. 2019). The tight junction between liver cells is around the bile duct, closing between cells and maintaining cellular polarity. The highly expressed tight junctions around the bile duct form a robust permeability barrier (Rao and Samak 2013). Their function is to prevent water and soluble entering or leaking from bile ducts. They constitute the biliary barrier. Disruption of this barrier function can lead to various biliary and liver diseases (Pradhan-Sundd and Monga 2019). However, the mechanisms by which the biliary barrier and tight junctions are impaired during ischemic injury remain largely unclear.

We previously reported that maintaining the hepatic artery flow open (HAFO) could improve the biliary ischemic injury, which may be associated with tryptophan metabolic pathway, as evidenced by differential expression of Kynureninase (Kynu), 3-hydroxyanthranilic acid dioxygenase (Haao), kynurenine aminotransferase 1 (Kat1) and Kat2 (Zhang et al. 2023). It is well-documented that de novo synthesis pathway from tryptophan could promote NAD biosynthesis, participate in metabolism, DNA repair, epigenetic regulation, senescence, and immunoregulation (Kurnasov et al. 2003; Minhas et al. 2019). Tryptophan catabolism is confirmed to be related to ischemia–reperfusion injury in various organs, including heart, brain, and kidney (Covarrubias et al. 2021; Katsyuba et al. 2020; Song et al. 2017; Kwiatkowska et al. 2020; Brouns et al. 2010). Tryptophan is primarily catabolized through the kynurenine pathway. Briefly, tryptophan is catabolized to kynurenine, which is then metabolized mainly by hydroxylation to 3-hydroxykynurenine (3-HK) via K monooxygenase (Kmo), followed by the hydrolysis of 3-HK to 3-hydroxyanthranilic acid (3-HAA) by Kynu. This enzyme can also hydrolyze hydrolyze kynurenic acid (KA) to anthranilic acid (AA) (Zulpaite et al. 2021). 3-HAA can undergo nonenzymic cyclization to quinolinic acid (QA), an agonist *of N*-methyl-d-aspartate (NMDA) receptor, which disrupts tight junction function in the blood brain barrier (Št’astný et al. 2000). Additionally, kynurenine and 3-HK are metabolized into kynurenic acid (KA) and xanthurenic acid (XA) by kynurenine aminotransferases (KATs). Study on intestinal epithelium demonstrates that XA and KA can maintain the integrity of the intestinal epithelial barrier and attenuated intestinal ischemia–reperfusion injury (Michaudel et al. 2023). Therefore, we propose that tryptophan metabolic pathway, particularly XA and QA can regulate tight junction and permeability of the biliary barrier, thereby improving bile duct injury.

Kynu, a member of the aminotransferase superfamily, is a key enzyme in the kynurenine pathway (Karamitros et al. 2020), which is involved in tryptophan metabolism. Our pervious data showed that Kynu expression was significantly upregulated in the more severe biliary ischemic injury group. This suggests that Kynu may play an crucial role in biliary ischemic damage. In this study, we report that Kynu-media kynurenine pathway is reprogrammed in response to hypoxia in vitro and ischemia-induced biliary system damage model. Inhibition of Kynu by shRNA or inhibitor can protect biliary system against ischemic injury by alleviating inflammation, restoring tight junction and reducing biliary barrier permeability.

## Materials and methods

### Animals

Male Sprague Dawley rats (180–220 g, 8–10 weeks old) were purchased from the Animal Experiment Center of Kunming Medical University (Kunming, China), maintained under controlled environmental conditions with a temperature of 22–24 °C, humidity of 55%–60%, 12 h light/12 h dark cycle, and permitted ad libitum access to standard rodent chow and water. All experiments were conducted in compliance with the Guide for the Care and Use of Laboratory Animals and were approved by the Animal Care and Use Committee of Kunming Medical University (License: kmmu2021058).

### Surgical procedures for short term

Rats were fasted overnight before operation and had free access to water. Anesthesia was carried out intraperitoneal injection of 10% chloral hydrate at a dose of 3 ml/kg. All surgical procedures were performed using microsurgery instruments (Shanghai Medical Instruments, Shanghai, China). In all groups, after a midline laparotomy, the ligaments around the liver were divided, the hepatic portal triad was isolated, and collateral vessels to the hepatic hilum were disconnected. The hepatic artery and portal vein were meticulously freed individually as previously described (Fouassier et al. 2007).

All animals were randomly divided into the following groups (6 animals per group) (Table 1):

**Table 1:** Definition of groups and detailed procedures

Groups	Portal vain	Hepatic artery
Sham	no intervention	no intervention
cPM	continuous occlusion for 45 min	continuous occlusion for 45 min
iPM	intermittent occlusion for (10 min occlusion/5 min opening) × 3	intermittent occlusion for (10 min occlusion/5 min opening) × 3
iHAFO	continuous occlusion for 45 min	Intermittent occlusion for (10 min occlusion/5 min opening) × 3
cHAFO	continuous occlusion for 45 min	continuous opening for 45 min
Mock	portal vein injection of 0.9% NaCl solution in cPM procedures	
BSZ	portal vein injection of benserazide hydrochloride (BSZ, 50 mg/kg) in cPM procedures	
shNC	50 nmol/100 g shRNA-NC were injected via tail vein 1 h before cPM	
shKynu	50 nmol/100 g shRNA-NC were injected via tail vein 1 h before cPM	

After 1 days and 1 weeks of reperfusion, animals were sacrificed. Blood samples were collected from the inferior vena cava were centrifuged at 4℃ and separated plasma was stored at − 80℃ for further biochemical analysis and amino acid test. Each liver lobe was taken and cut into two pieces through the middle with a razor blade. One piece was put into liquid nitrogen for molecular analysis; the other piece was fixed in 4% neutral buffered formaldehyde for histological studies. The extrahepatic bile duct was taken 5 mm near the hepatic hilum and divided into two parts by a blade, one part was put into 2.5% glutaraldehyde in cacodylate buffer and 1% OsO_4_ for electron microscopy, and the other part was sampled in 4% neutral buffered formaldehyde for further analysis.

### Liquid chromatography-tandem mass spectrometry

Protein was precipitated by the addition of ice-cold 100% trichloroacetic acid to samples, followed by incubation for 30 min at 4 ℃ and centrifugation to obtain the supernatant. Serial dilutions of each metabolite were prepared over appropriate concentration ranges to prepare a calibration curve to permit quantitation. In all, 10 µL volumes of each sample were injected onto a Waters Select HSS XP column (30 mm × 100 mm, 2.5 µm, Waters Corp, Elstree, Herts) using a Waters Acquity UPLC autosampler, coupled to an ABSciex QTRAP 5500 mass analyzer. The flow rate was 0.35 mL/min at 25 ℃. Data were acquired and processed using Analyst quantitation software (ABI Sciex).

### Histological, immunohistochemistry analysis of extrahepatic and intrahepatic bile ducts injury

Common bile duct and liver tissue were fixed in 4% neutral buffered formaldehyde, paraffin-embedded, cut into 4 μm thickness, and stained with hematoxylin and eosin (H&E). Histological images were captured by DM6000B (Leica, Germany). Bile duct injury was assessed with the methods previously described (Reiling et al. 2017).

For the immunohistochemistry analysis, 4-μm thick paraffin-embedded tissue sections were deparaffinized in xylene, rehydrated in graded alcohol and blocked in methanol containing 3% hydrogen peroxide. The slides were covered with a blocking solution for 1 h at room temperature and incubated with rabbit anti-MPO antibody or rabbit anti-Ki67 antibody for 2 h at 37 °C. After rinsing with phosphate-buffered saline (PBS; pH 7.4) solution, sections were treated with a goat anti-rabbit secondary antibody (Santa Cruz Biotechnology, Santa Cruz, CA, USA) for 1 h at 37 °C. Next, the slides were incubated with 3,3-diaminobenzidine (DAB) solution for 10 min and then counterstained with hematoxylin. Ki67 and MPO expression were quantified using DM6000B (Leica, Germany). The primary antibody was in Table S2.

### Transmission electron microscopy

Transmission electron microscopy was carried out as previously described (Fickert et al. 2002). The fixed extrahepatic bile ducts were infiltrated with acetone-araldite, and embedded in araldite. Then, 80 nm sections were cut, treated with uranyl acetate and lead nitrate. The slices were exanimated with the transmission electron microscope (JEOL Ltd., Japan).

### Biochemical analysis of serum

Serum alanine aminotransferase (ALT), aspartate aminotransferase (AST), alkaline phosphatase (AKP), gamma-glutamyl transferase (GGT), total bilirubin (TBIL), and direct bilirubin (DBIL) were tested by a colorimetric assay with COULTER AU480 (Beckman Coulter, CA, USA).

### Cytokine array and LDH of bile

Lactate dehydrogenase (LDH) in bile was measured via colorimetric assay (#5604–01, Bioo Scientific Corporation, Austin, TX). The concentrations of Interleukin-6 (IL-6), Interleukin-10 (IL-10), Tumor necrosis factor-α (TNF-α), Monocyte Chemoattractant Protein-1 (MCP-1) in the bile were assessed using ELISA assay (Neobioscience, China).

### TUNEL/CK19 and ZO-1/CK19 double immunofluorescence (IF) staining

Formalin-fixed liver sections were deparaffinized and pretreated with proteinase K followed by horseradish peroxidase TdT-mediated dUTP nick end labeling (TUNEL) using the In Situ Cell Death Detection Kit (Roche Diagnostic, Germany). For double labeling, TUNEL-stained sections were boiled in a water bath with 10 mM citric acid, pH 6.0, for 20 min, blocked with normal horse serum, and incubated with anti-cytokeratin 19 (CK19) (1:50; Proteintech, China) for 60 min. For the latter, visualized with goat anti-rabbit IgG antibodies conjugated with Alexa Fluor Dyes (red stained) (Invitrogen) by confocal laser scanning microscopy (LSM5 Pascal Exciter) (original magnification, × 400). For quantitative analysis, we counted TUNEL + hepatocytes and cholangiocytes (CK19 stained) in 10 randomly selected fields around the bile infarcts per section (original magnification, × 400).

When ZO-1 and CK19 were costained, the ZO-1 antibody (1:100; Proteintech, China) was applied first as described above, followed by incubation with a fluorescently labeled secondary antibody. Then, blocking with 10% donkey serum for 60 min was repeated, and goat anti-rabbit IgG was applied after blocking, followed by washing with PBS. Afterwards, the CK19 antibody (1:50; Proteintech, China) was applied routinely, followed by incubation with another fluorescently labeled secondary antibody.

### Indian ink or horseradish peroxidase (HRP) injections and detection

Rats in each group were randomly select, and were performed retrograde low-pressure injection of Indian ink into the bile duct. Bile duct near the liver hilum were exposed, then were injected 8 mL/h of Indian ink (Oddfoni Co. Ltd., Nanjing, China) before histological examination as reported in previous studies (Jiang et al. 2016).

Horseradish peroxidase (HRP, Peroxidase from horseradish type II, P8250, Sigma-Aldrich, St. Louis, MO, USA) was used to assess the permeability of the BBB in vivo. Two hundred microliter of HRP (5000 IU/mL in sterile water) was injected into the inferior vena cava of each animal 30 min after cannulation of the common bile duct. Bile was subsequently collected for 10 min to assess paracellular transport across tight junction. A commercially available kit (#A22188, Thermo Fisher Scientific, Waltham, MA, USA) was used to determine activity of HRP in bile.

### Cell culture for cholangiocyte and treatment

The in vitro experiments were performed in a normal human cholangiocyte cell line (H69, LONZA, Switzerland). Cholangiocyte were maintained in culture with DMEM/F-12 (Gibco, Waltham, MA, USA), supplemented with 10% (v/v) fetal bovine serum (FBS) and 1% (v/v) penicillin/streptomycin (P/S) (both from Thermo Scientific, Waltham, MA, USA). All cell lines were cultured at 37 °C under 5% CO2 in a humidified incubator.

Cholangiocytes were seeded in six-well plates and allowed to attain 70–75% confluence. The cells were then incubated in serum-free and antibiotic-free medium for 24 h and transfected with transfected with the shKYNU lentivirus, involving KYNU mRNA knockdown and the empty vector lentivirus shNC (Tsingke Biotechnology, China). Control RNA and shRNAs were complexed with Oligofectamine Plus reagent and Fugene-6 and added to the cell monolayers and incubated for 6 h. Serum (FBS) level was restored to 10% and the cells were allowed to stabilize for 20 h.

Similar, after 24 h culture, cholangiocyte were incubated with BSZ (10 µM; Cayman chemical company, USA), QA (100 µM), XA (100 µM) or AA (100 µM) for 24 h. Then cell monolayers were then trypsinized and seeded onto Transwell inserts for further experiments.

### Hypoxia and reoxygenation stimulation

The cells were seeded in Petri dishes and maintained in their medium. Cells were then placed in the incubator at 37 °C and exposed to hypoxia in a modular incubator chamber (C-Chamber Hypoxia chamber) with continuous monitoring and automated adjustments to maintain the several parameters stable (ProOx controller; Biospherix, Redfield, NY). Cholangiocyte were maintained under standard conditions in normoxic atmosphere of 21% O2, 74% N2, and 5% CO2 to reflect physiologic conditions or under a hypoxic atmosphere of 5% O2, 90% N2, and 5% CO2 for 24 h and transferred to normal conditions for 24 h.

Afterward, we extracted from cholangiocyte proteins and total RNA to measure changes in 1) cell proliferation by CCK8; 2) levels of Interleukin-6 (IL-6), Interleukin-10 (IL-10), Tumor necrosis factor-α (TNF-α), Monocyte Chemoattractant Protein-1 (MCP-1) by ELISA assay; 3) tight junction by Immunofluorescent staining for ZO1; 4) expression of ZO1, Occludin, GluR2 and CX50 by qPCR oand immunoblotting. An analysis was performed using cholangiocyte maintained at normal levels of oxygen for 24 h as the control sample.

### Cell viability assay

Cell counting kit-8 (CCK8, Beyotime Institute of Biotechnology, Shanghai, China) was usedto test the viability of Cholangiocytes. Cholangiocytes transfected with shKynu or BSZ (4000 cells per well) were seeded in 96-well plates. After cell adherence, 10 μL of CCK-8 solution were added in each well and cells were maintained in a5% CO2 incubator at 37 °C. After 1 h, OD values at450 nm were recorded. All groups were replicated for 5 wells.

### Cytokine array in cell culture supernatants

The concentrations of Interleukin-6 (IL-6), Interleukin-10 (IL-10), Tumor necrosis factor-α (TNF-α), Monocyte Chemoattractant Protein-1 (MCP-1) in the cell culture supernatants were assessed using ELISA assay (Neobioscience, China).

### Measurement of transepithelial electrical resistance (TEER)

TER was measured as described before (Vanuytsel et al. 2021) by using a Millicell-ERS electrical resistance system (Millipore, Bedford, MA). The TER was recorded in empty Transwell inserts (usually 50–80 Ω·cm2) and was subtracted from all values.

### Inulin permeability

Mature cell monolayers on Transwells were incubated in different experiments with 0.5 mg/ml FITC-inulin in the apical chamber. At the end of incubation, 100 μl each of basal medium were sampled, and fluorescence was measured with an Infinite® 200 PRO microplate fluorescence reader (TECAN, Switzerland). Flux of FITC-inulin into the basal well was calculated as the percentage of total fluorescence administered into the apical well per hour per square centimeter of surface area.

### Immunofluorescence for cells

Microscopy Under various experimental conditions cell monolayers were fixed in acetone-methanol (1:1, vol/vol) at 0 °C for 5 min. The fixed membranes were rehydrated in PBS and permeabilized with 0.2% Triton X-100 in PBS. Cell monolayers were blocked with 4% nonfat milk in Tris-buffered saline containing 0.05% Tween-20 for 1 h at room temperature. After overnight incubation with the primary antibody for ZO-1 (1:200, Proteintech, China) at 4 °C, the cells were washed three times with PBS, and incubated with AlexaFluor-488-conjugated anti-rabbit IgG secondary antibodies (1:500, BioLegend) for 1 h in the dark at room temperature. The cell nuclei were stained with DAPI (1:5000, Sigma) followed by three times washing with PBS. Cells were mounted and images were collected using a Zeiss LSM 5 PASCAL laser scanning confocal microscope with LSM 5 PASCAL software (Release 3.2). A series of images was collected from 1.0-μm XY sections. Images were stacked by use of the Image J software and processed by Adobe Photoshop (Adobe Systems, San Jose, CA).

### RNA extraction and real-time PCR

Total RNA was extracted from 1 × 10^5^ cells or 50 mg liver tissue using the Trizol reagent (Invitrogen) and reverse transcribed using RevertAid first-strand cDNA synthesis kit (Takara, Dalian, China). Quantitative real-time polymerase chain reaction (qRT-PCR) was performed using the QuantStudio™ 12 K Flex Real-Time PCR System (Applied Biosystems, Life Technologies Corp., CA, USA). Beta-actin was used as an internal control. Primer sequences of Kmo, Kynu, Kat, Haao were listed in Table S1. The relative mRNA expression was analyzed by the comparative cycle threshold (Ct) method. All experiments were performed in triplicate.

### Western blotting

Western blotting was performed as described in previous study (Huang et al. 2010). Total liver protein were extracted and separated in 10% SDS-PAGE and transferred to polyvinylidene difluoride membranes (Millipore Corporation, Billerica, MA, USA). The membranes blocked in Blocking Buffer (Beyotime, China) for 1 h, and then incubated overnight at 4℃ with the primary antibodies. After incubated with secondary antibody, and β-actin was used as an internal control, the protein expression was analyzed by the comparative β-actin using gray level difference analysis. Primary antibodies were listed in Supplementary Table S2. All experiments were performed in triplicate.

### Statistical analysis

IBM SPSS software (Version 22.0) was used for the statistical analysis. Continuous values were expressed in means and standard deviations when normally distributed while in medians and interquartile ranges when not normally distributed. For normally distributed values, the ANOVA test with Bonferroni correction for post hoc pairwise t-tests were used. For non-normally distributed values, the Kruskal–Wallis test and Mann–Whitney test were employed. A per-protocol analysis was conducted. The significance level for statistical hypothesis-testing procedures concerning comparisons of means was set at *p* < 0.05, and all tests were two-tailed.

## Results

### Transcriptome and proteome analysis revealed the relationship between tryptophan metabolism pathway and biliary ischemia injury

**Fig. 1:**
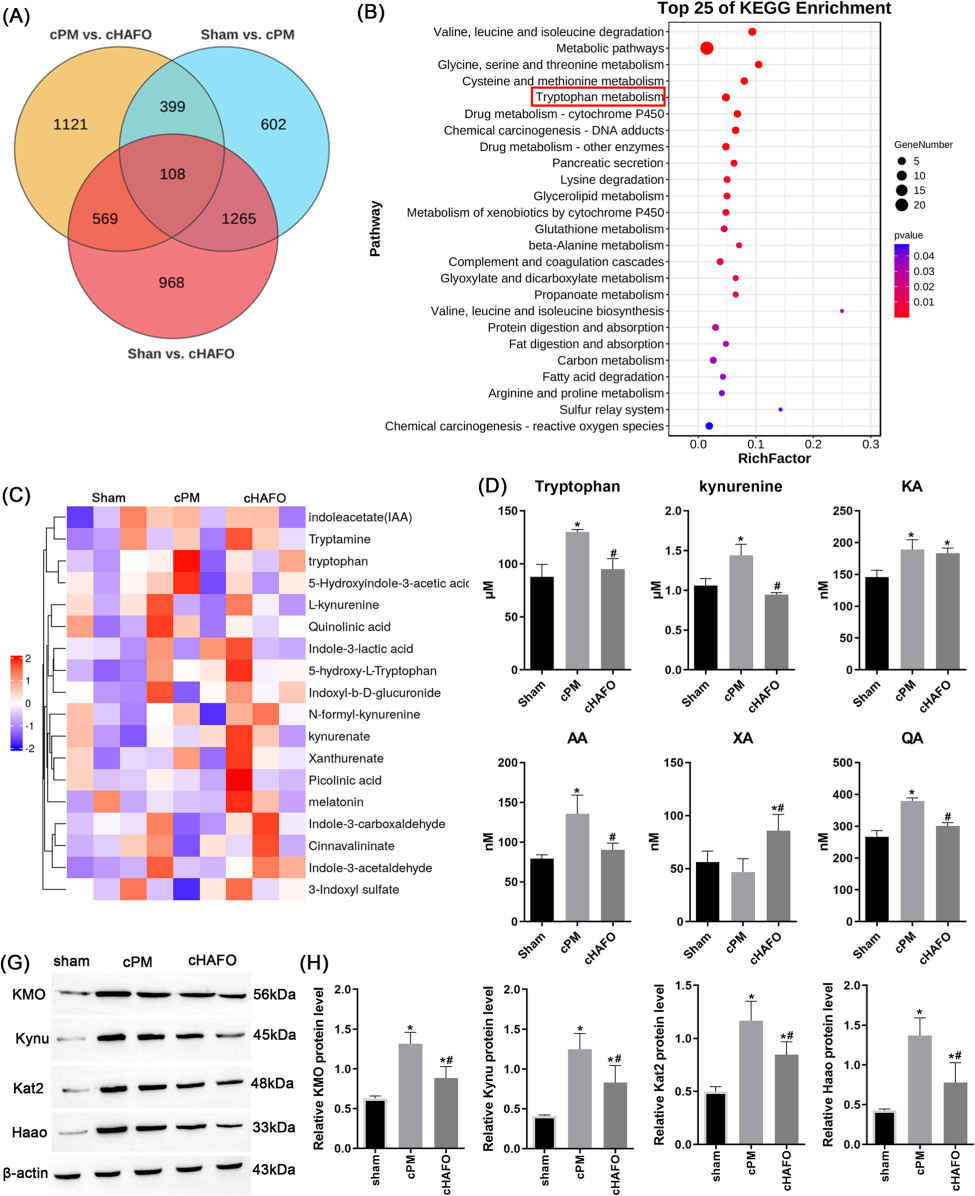
Transcriptome and proteome analysis between different types of hepatic blood inflow occlusion (biliary ischemia injury). **A** Venn diagram was applied to present the co-DEGs among sham, cPM and cHAFO group. **B** KEGG term enrichment analysis of co-DEGs between cPM and cHAFO groups. **C** Differential analysis of the abundance of TRP metabolites in the bile between cPM and cHAFO groups. **D** Relative abundance of individual metabolite between different groups, including tryptophan, L-Kynurenine, KA, XA, 3-HAA, and QA in the bile of rats subjected to 45-min ischemia followed by reperfusion for 24 h. *n* = 6 rats for each group. **G** Detection of Trp-QA metabolic pathway (by immunoblotting analysis for Kmo, Kynu, Kat2, Haao). **H** Relative expression of Kmo, Kynu, Kat2, Haao in the livers of rats from different group. *n* = 6 rats for each group. * *P* < 0.05 vs. sham, #*P* < 0.05 vs. cPM

To investigate the alteration of transcriptomic profiles during bile duct ischemic injury, RNA sequencing was performed using extrahepatic bile ducts tissue samples from rats subjected to sham, cPM (severe bile duct ischemia injury) and cHAFO (mild bile duct ischemia injury). A total of 108 co-differential expressed genes (DEGs) were identified in the isolated extrahepatic bile duct tissue (FoldChange > 1.5, *p* < 0.05; Table S3, Fig. 1A). Among these DEGs, several genes associated with tryptophan metabolism, including Kynu and Kyat3, were differential expressed. KEGG pathway analysis revealed enrichment of 26 metabolic pathways across the sham, cPM, and cHAFO groups, including Tryptophan metabolism, Retinol metabolism, Ascorbate and aldarate metabolism, Steroid hormone biosynthesis, Histidine metabolism, Arachidonic acid metabolism, Glycine, serine and threonine metabolism (Fig. 1B, Table S4).

Tryptophan is metabolized through three major pathways: the serotonin pathway, the kynurenine pathway and the indole pathway. The kynurenine pathway has been widely implicated in the inflammatory process, contributing to ischemic injury. We performed targeted quantitative metabolomics approach to measure tryptophan metabolites in bile (Fig. 1C). Compared to sham group, elevated levels of AA and QA were observed in the cPM group, whereas XA levels decreased and KA remained unchanged (Fig. 1D). In contrast, XA levels increased in cHAFO group. Compared with cPM group, cHAFO group showed reduced levels of Tryptophan, Kynurenine, AA and QA.

To elucidate the regulatory mechanism underlying tryptophan metabolic reprogramming during hepatic ischemia injury, we assessed the expression of key enzymes involved in the kynurenine pathway. RNA-sequencing data indicated upregulated expression of KMO, Kat2, Kynu and Haao. Immunoblotting confirmed that cPM increased the protein levels of Kynu, Haao and Kat2/Aadat, but did not alter Kmo expression (Fig. 1E-F). Compared to cPM group, mild bile duct injury in the cHAFO group was accompanied by a downward trend in hepatic expression of Kynu, Kat2, Kmo and Haao (Fig. 1E-F). The pronounced upregulation of Kynu, consistent with our RNA-seq findings, may explain the significant elevation in Trp-QA levels observed above.

### Kynu inhibition extenuated hypoxia-induced tight junction dysregulation and inflammation in biliary epithelial cell

**Fig. 2:**
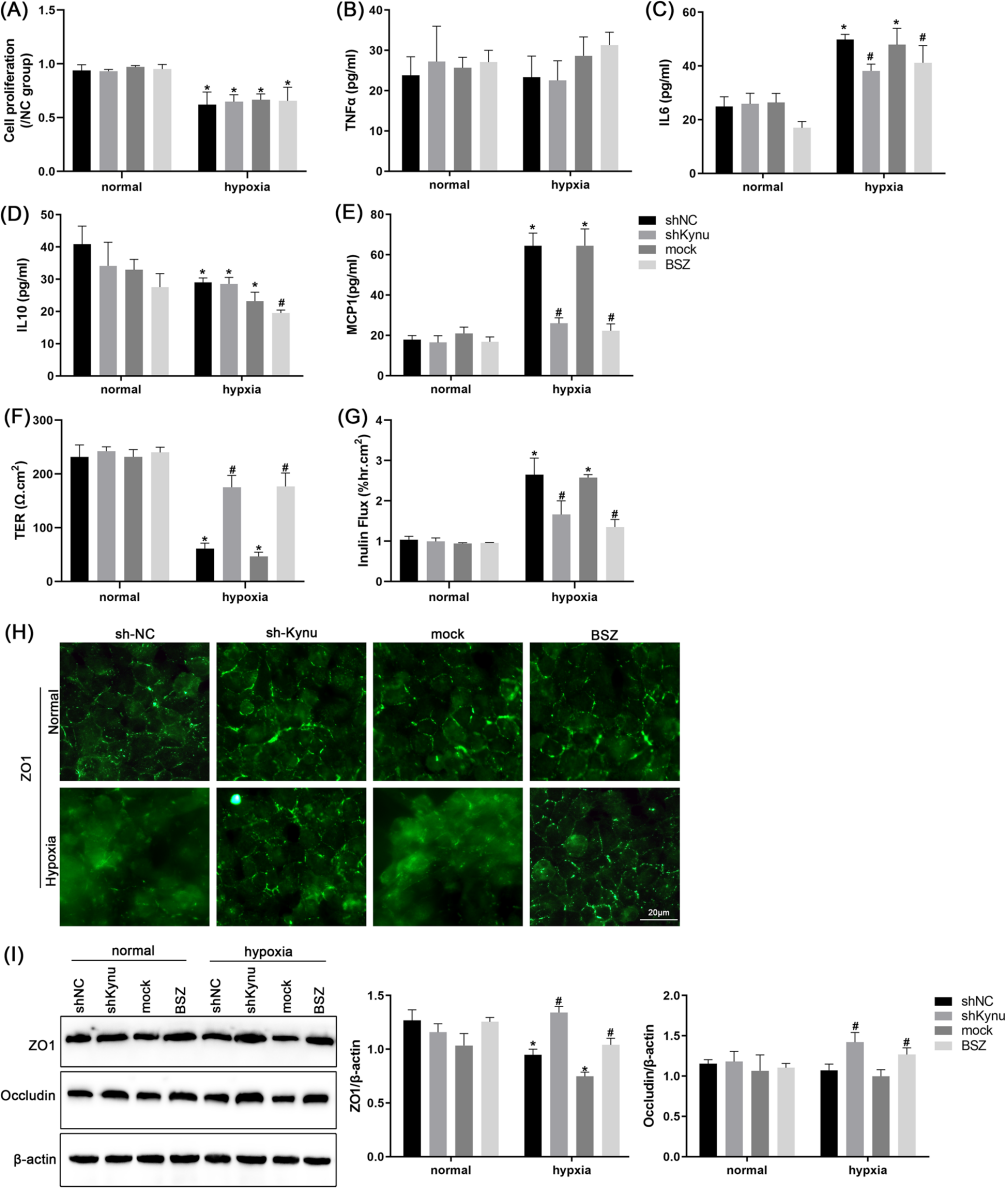
In vitro effect of Kynu inhibition extenuated hypoxia-induced cell proliferation, tight junction dysregulation and inflammation. **A** Evaluation of cell proliferation in normal rat extrahepatic cholangiocyte cultures maintained in condition of normoxia or hypoxia for 4 h and transferred to normal conditions for 1 h with or without pre-incubation with Kynu shRNA or BSZ. **B**-**E** Inflammatory factor was investigated by ELISA in the same in vitro samples, including TNFα, IL-6, IL-10 and MCP1. **F** Barrier function and **G** inulin permeability were measured on day 2 after seeding. **H** Polarized cholangiocyte monolayers were stained with the indicated antibodies and imaged by confocal microscopy. green = ZO1, blue = DAPI, signal. Insets: 2.5 × magnification. Scale bars = 20 µm. **I** The protein levels of ZO1 and Occludin were measured by immunoblot analysis. Data are means ± S.D. of 3 blots. *P < 0.05 vs. its corresponding control value; #*P* < 0.05 vs. normoxia control value

We hypothesized that cPM might influence cell oxidative-stressed damage in ischemia-stressed duct, given Kynu’s established role in the maintaining cellular redox homeostasis. Thus, we examined the effect of Kynu knockdown on cholangiocyte response to hypoxia (Fig. S1). shKynu did not affect cholangiocytes proliferation compared to normoxia or hypoxia treated cells (Fig. 2A). We also examined the change of inflammatory factor levels, including IL-6, IL-10, TNF-α, and MCP-1 (Fig. 2B-E). Hypoxia significantly increased IL-6 and MCP-1 secretion while reduced IL-10 level. There is no statistical difference in TNF-α levels between normoxic and hypoxic conditions. Neither shKynu nor BSZ (A Kynu inhibitor) altered these factors under normoxia, but both reduced the IL-6 and MCP-1 level under hypoxia. No changes in IL-10 and TNF-α with shKynu and BSZ treatment were observed during hypoxia. Barrier function was assessed by measuring TEER and inulin permeability in hypoxic cholangiocyte monolayers. Hypoxia reduced TER and increased inulin permeability (Fig. 2F-G), which could be attenuated by shKynu or BSZ. Immunofluorescence staining revealed disrupted tight junction (TJ) protein localization under hypoxia (Fig. 2H). Notably, Kynu knockdown prevented this disruption independently of changes in TJ protein mRNA transcripts or protein levels (Fig. 2I). These findings demonstrate that Kynu inhibition reduces paracellular permeability and protect cells against hypoxia-induced ischemic injury in vitro.

### Kynu inhibition ameliorated cPM-induced bile duct injury in vivo

**Fig. 3:**
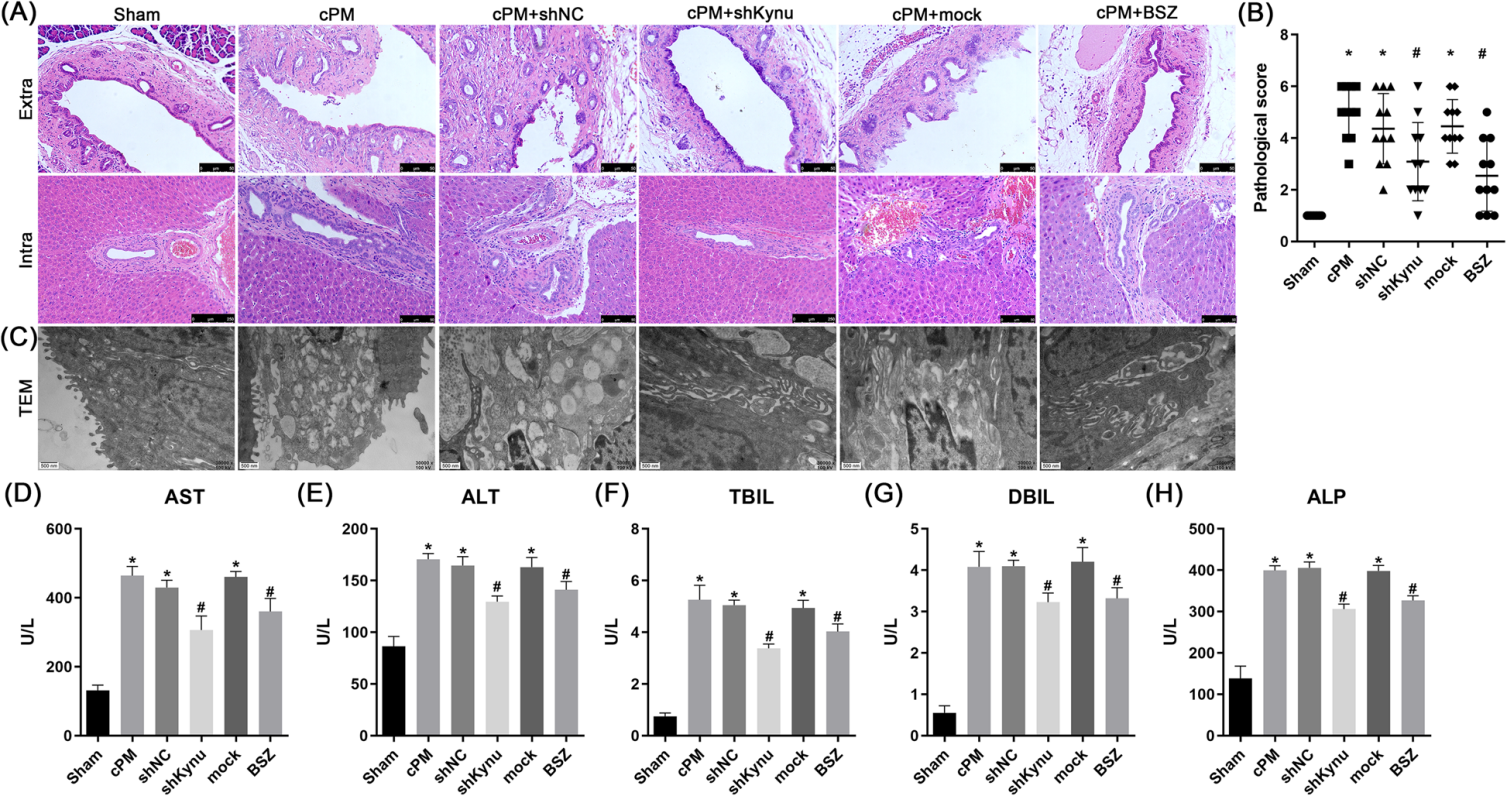
Kynu inhibition attenuates bile duct injury after cPM treatment. **A** Histopathological changes in extra-hepatic and intra-hepatic bile ducts 24 h after cPM treatment (HE × 400). More edematous, necrotic and deciduous biliary epithelial cells are observed in the cPM group compared with the shRNA or BSZ group. Bile duct tissue in the Sham group presents a normal histopathological architecture. **B** The pathological score in the cPM group is much higher than that in the shRNA or BSZ group. The score was determined by manual counting in 10 random visual fields (× 400). **C** Changes in bile duct in the sham, cPM, shNC, shRNA, mock and BSZ groups were observed by TEM. **D**-**H** The ALT, AST, TBIL, ALP and DBIL levels in the sham, cPM, shNC, shRNA, mock and BSZ groups at 24 h after IRI. *n* = 6 rats for each group. Values are expressed as the mean ± SD. Data from a representative (*n* = 3) experiment are shown. *P < 0.05 vs. sham group; # *P* < 0.05 vs. cPM group

Next, we investigated the effect of Kynu inhibiting via shRNA or BSZ on bile duct injury in the rat ischemic model. Neither shKynu nor BSZ treatment induced mortality in rats, regardless of hepatic ischemia. Compared to sham group, cPM group exhibited more severe intra-hepatic and extra-hepatic bile duct damage. Intraperitoneal administration of shKynu or BSZ significantly attenuated intra-hepatic and extra-hepatic bile duct injury, as evidenced by reduced number of edematous, necrotic biliary epithelial cells with deciduous morphology relative to the cPM group (Fig. 3A). A bile duct injury severity score (BDISS) was employed for quantitative assessment. Histological evaluation revealed significantly lower BDISS values in shKynu- and BSZ-treated groups compared to the cPM group (Fig. 3B). TEM analysis confirmed these findings (Fig. 3C). Serum levels of ALT, AST, ALP, TBIL and DBIL showed significant reduction following shKynu or BSZ treatment, demonstrating improvement in cPM-aggravated liver and bile duct pathology (Fig. 3D-H). These data collectively indicate that inhibiting Kynu could attenuate cPM-induced ischemic injury in the bile duct.

### The effect of Kynu inhibition on cholangiocytes apoptosis, proliferation and neutrophil infiltration in vivo

**Fig. 4:**
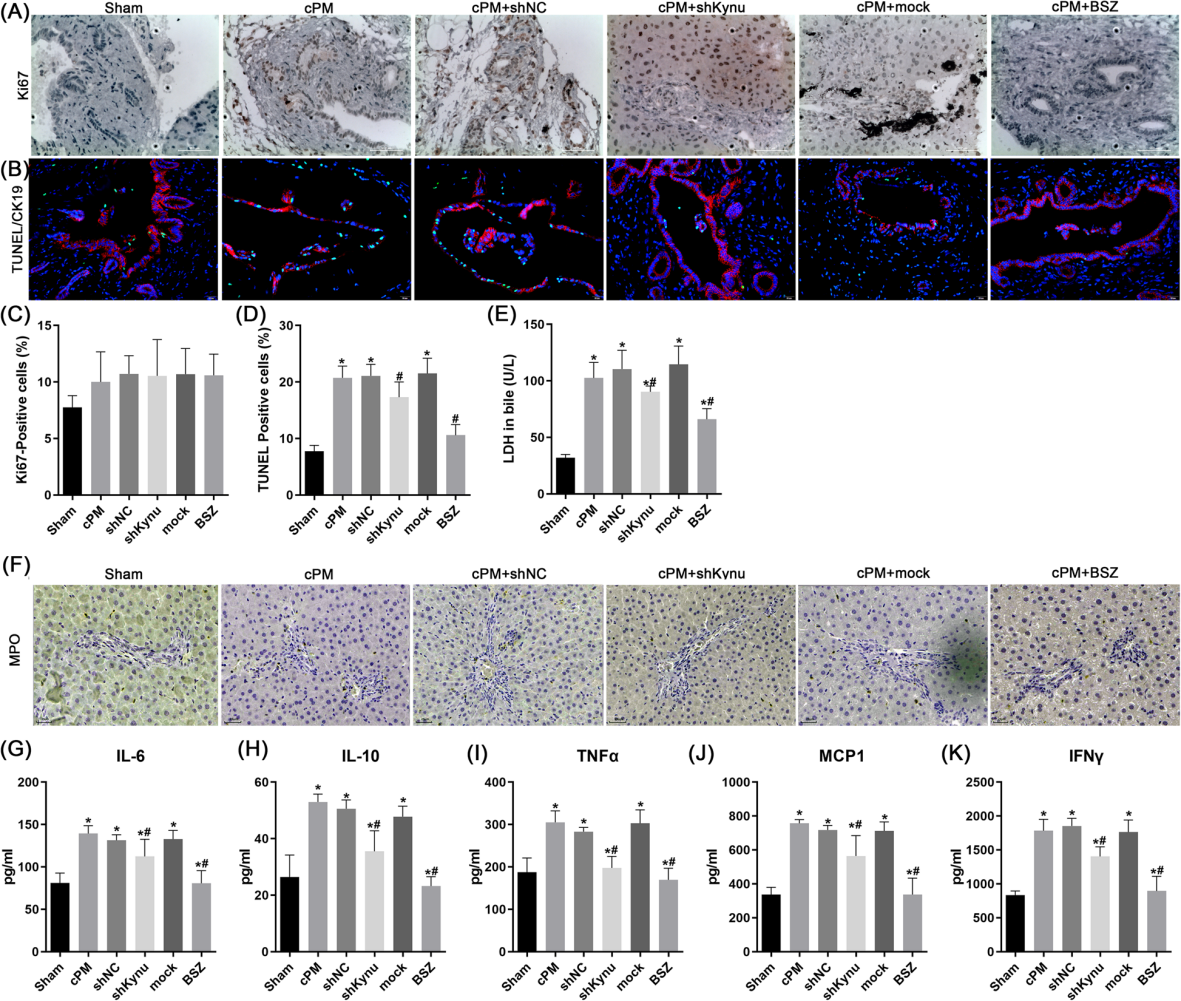
Kynu inhibition prevents IR-induced cholangiocytes apoptosis and proliferation, and suppresses the inflammatory response in bile duct tissue after reperfusion. Representative light microscopy images of bile duct sections (200 × magnification) stained for Ki67 (**A**) and TUNEL DNA fragmentation (TUNEL assay) (**B**). Quantification of Ki67 + (**C**) and TUNEL + cells (**D**) per high-powered field. (**E**) LDH levels in bile. **F** lmmunohistochemistry of liver for MPO, inflammatory cells are infiltrating the small bile ducts as neutrophils are stained brown (× 200). **G**-**K** The levels of IL-6, IL-10 m TNF-α, IL-10, MCP-1 and IFNγ in bile was determined by ELISA at 24 h after cPM treatment. The results are depicted as the mean ± SD (*n* = 3). *n* = 6 rats for each group. Data from a representative (*n* = 3) experiment are shown. *P < 0.05 vs. sham group; # *P* < 0.05 vs. cPM group

We further investigated the effect of Kynu inhibition on cholangiocytes proliferation and apoptosis. Biliary injury may trigger a proliferative response (ductular reaction). The proportion of Ki67-positive biliary epithelial cells increased in cPM group but remained unchanged following shKynu or BSZ treatment (Fig. 4A and C). Apoptosis of biliary epithelial cells was determined by TUNEL assay at 24 h after cPM treatment. Compared to Sham group, a higher number of TUNEL-stained intra-hepatic and extra-hepatic bile duct cells was observed in the cPM groups, which was attenuated by BSZ or shKynu treatment (Fig. 4B and D). Additionally, shKynu or BSZ reduced cPM-induced LDH activity in bile, a biomarker for biliary injury (Fig. 4E).

Next, we measured the cytokines in bile to evaluate the effect of Kynu inhibition on the local inflammatory milieu surrounding cholangiocytes. The MPO-positive cells infiltrating into the bile duct were increased significantly in the cPM group, indicating that neutrophil infiltration resulting from bile duct damage (Fig. 4F). Treatment with shKynu or BSZ completely prevented neutrophil infiltration, as number of MPO-positive cells of this group matched those of sham-operated rats (Fig. 4F). Bile levels of proinflammatory cytokines, including IL-1, IL-6, TNF-α, and MCP-1, were significantly elevated in the cPM group compared to sham group. Following Kynu inhibition, pro-inflammatory cytokines levels were substantially reduced (Fig. 4G-K). These data showed that the protective effect of Kynu inhibition was associated with diminished cytokine and reduced inflammatory cell infiltration.

### Down-regulation of Kynu restore tight junction and protects the integrity of biliary-barrier following ischemia in vivo

**Fig. 5:**
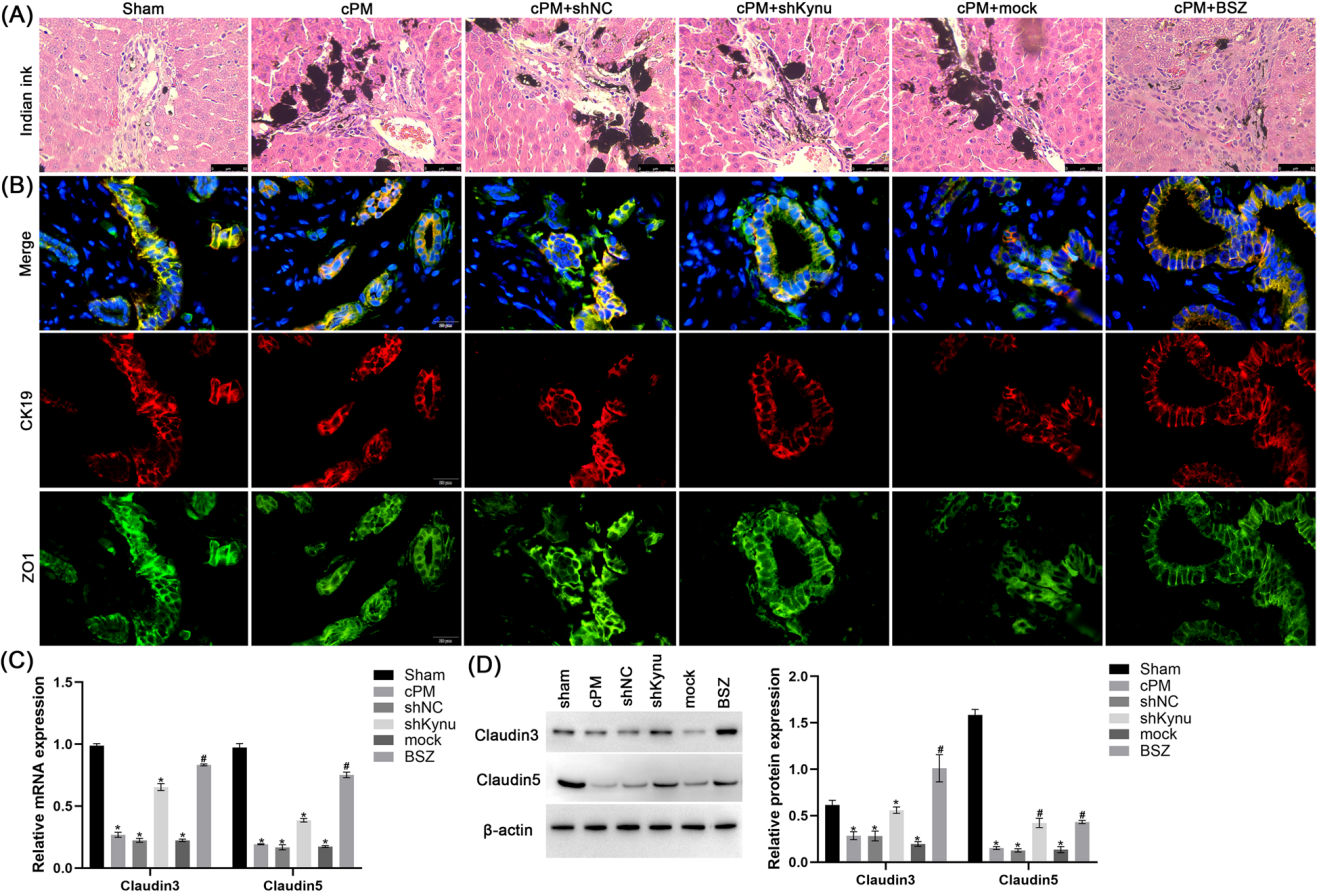
Effect of Kynu inhibition on biliary-barrier and tight junction in vivo. **A** Changes of the integrity of biliary-barrier present as India ink leakage following ischemia. **B** Changes in ZO-1(tight junction protein, green)/CK19 (cholangiocytes; red)/DAPI (nuclei, blue) expression in the sham, cPM, shNC, shRNA, mock and BSZ groups were observed under a fluorescence microscope. **C** The relative mRNA levels of Claudin-3 and Claudin-5 expression were calculated as a ratio to β-actin gene expression. **D** Western blot images illustrate the protein expression of Claudin-3 and Claudin-5 in each group. *n* = 6 rats for each group. The results are depicted as the mean ± SD. Data from a representative (*n* = 3) experiment are shown. *P < 0.05 vs. sham group; # *P* < 0.05 vs. cPM group

Previous studies have demonstrated that cPM induces inflammatory factors, stimulates Kynu (secrete Try\Kynurenine, etc.), damages the bile duct cells and disrupts the biliary barrier, ultimately leading to complications in liver transplantation (Kochhar et al. 2013; Campbell et al. 2014; Krupa and Kowalska 2021). Therefore, we investigated the effect of Kynu inhibition on biliary-barrier function. Compared to the Sham group, cPM significantly increased biliary permeability, as evidenced by elevated output of intravenously administered indian ink in bile. This effect was ameliorated by either shKynu or BSZ administration (Fig. 5A). Immunofluorescence staining revealed intact ZO-1/CK19 localization at cholangiocyte junctions in sham-operated rats without biliary injury (Fig. 5B). By contrast, cPM-treated, shNC, and mock groups exhibited disrupted tight junction morphology with irregular ZO-1/CK19 staining patterns (Fig. 5B). qPCR analysis demonstrated altered mRNA expression of tight junction proteins, including Claudin-3 and Claudin-5 (Fig. 5C). Immunoblotting confirmed that shKynu or BSZ treatment upregulated Claudin-3 and Claudin-5 protein levels, which decreased in cPM group (Fig. 5E). Collectively, these findings indicate that Kynu inhibition preserves biliary barrier integrity following ischemia.

### Down-regulation of Kynu disrupted tryptophan metabolism pathway, particularly by increasing XA and decreased QA levels

**Fig. 6:**
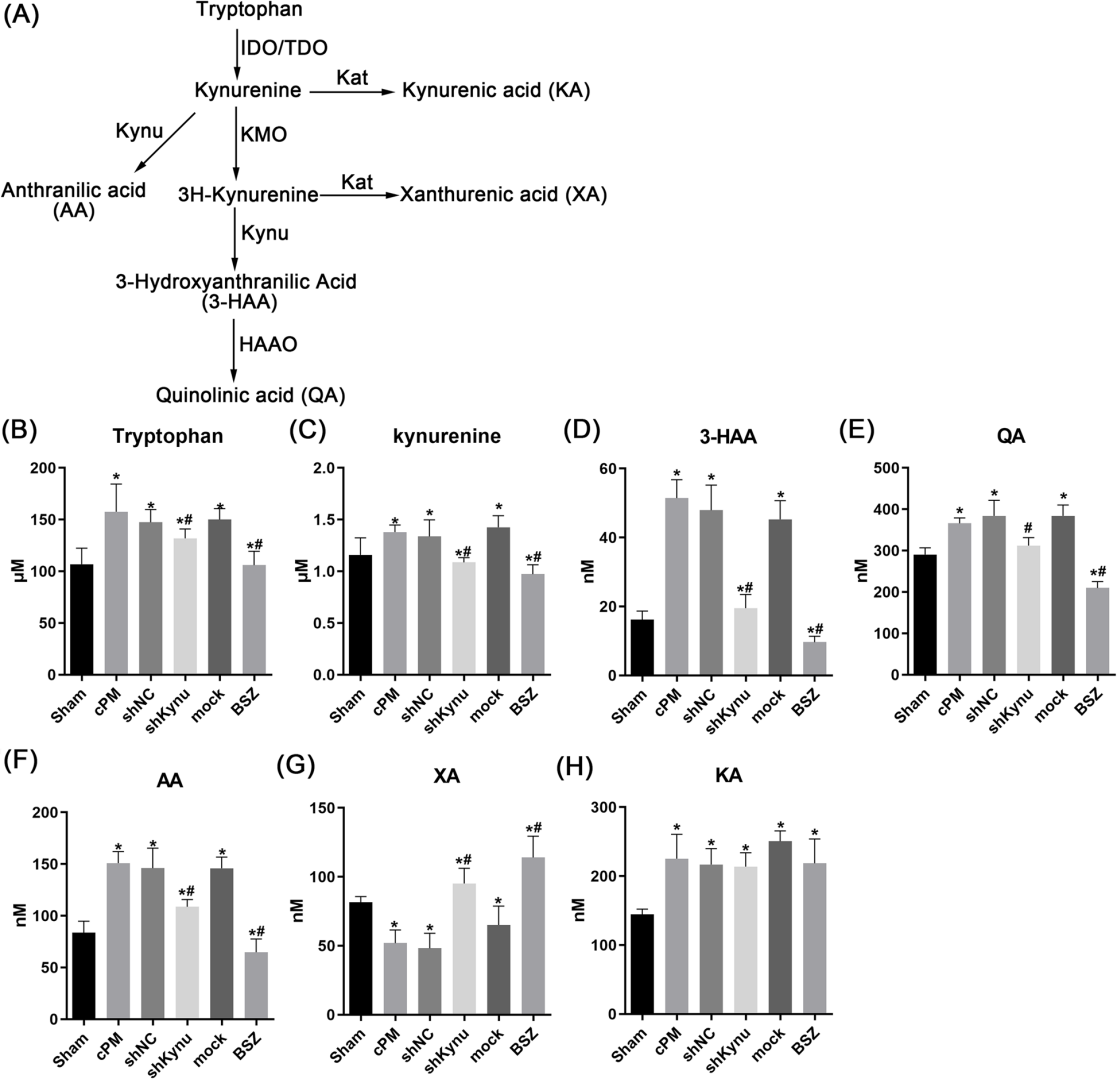
Down-regulation of Kynu disregulated tryptophan metabolism pathway, especially increased the levels of XA and decreased QA. **A** changes of Kynurenine pathway. **B** Tryptophan, kynurenine, 3-HAA, QA, AA, XA and QA levels in liver tissue from rats treated with cPM alone or with Kynu shRNA or BSZ. *: *p* < 0.05, KA, kynurenic acid; XA, xanthurenic acid

Several tryptophan metabolism products may be associated with cholangiocyte homeostasis, potentially contributing to bile duct injury. Therefore, we i examined alterations in metabolites from the kynurenine pathway following Kynu inhibition (Fig. 6A). Compared to the sham group, liver concentrations of Trp and Kyn were elevated in the cPM group. This effect was reversed by shKynu or BSZ treatment (Fig. 6B-C). We further assessed the expression of key enzymes in this pathway. Hepatic gene expression of Kynu and Haao significantly increased following shKynu or BSZ administration, concomitant with reduced liver concentrations of 3-HAA, QA and AA (Fig. 6D-F). Conversely, XA levels were elevated upon shKynu or BSZ treatment, correlating with upregulated Kat2 expression (Fig. 6G). Notably, neither intervention altered Kmo expression and KA levels (Fig. 6H).

These data suggested that Kynu inhibition reprograms the Trp-QA pathway, leading to metabolic shifts—particularly an increase in XA and a decrease in QA levels.

### The effect of Kynu on tight junctions may be mediated through XA regulating glutamate receptors 2/3 (GluR2/3) and Connexin 50 (CX50) expression

**Fig. 7:**
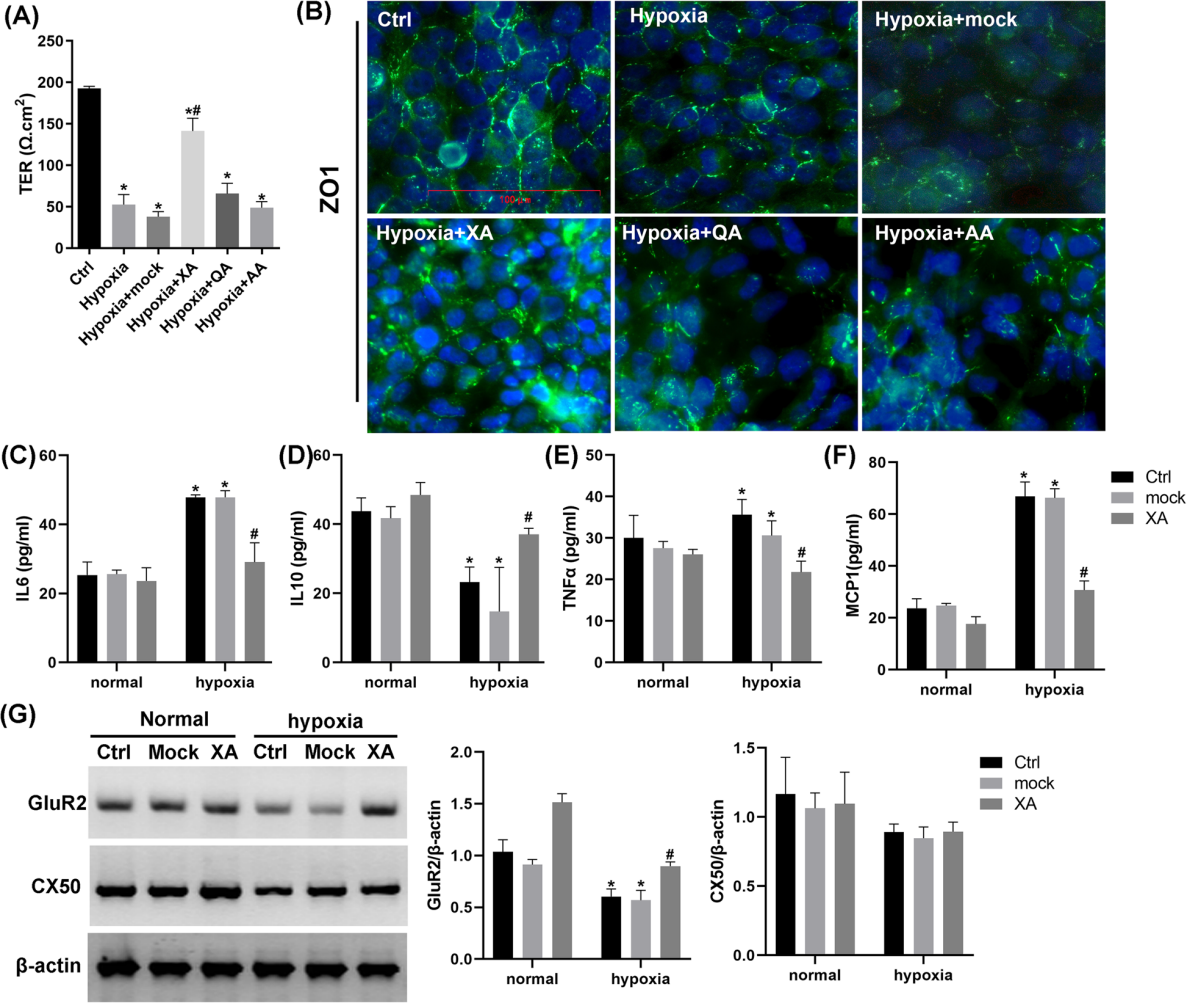
XA regulated glutamate receptors 2/3 (GluR2/3) and Connexin 50 (CX50) expression improve impaired tight junction and inflammation in vitro. Normal rat extrahepatic cholangiocyte cultures maintained in condition of normoxia or hypoxia for 4 h and transferred to normal conditions for 1 h with or without pre-incubation with XA. **A** Barrier function were measured on day 2 after seeding. **B** Polarized cholangiocyte monolayers were stained with ZO1 (green)/DAPI (blue) and imaged by confocal microscopy. Scale bars = 100 µm. **C**-**F** Inflammatory factor was investigated by ELISA in the same in vitro samples, including TNFα, IL-6, IL-10 and MCP1. **G** The protein levels of GluR2/3 and CX50 were measured by immunoblot analysis. Data are means ± S.D. of three experiments. *P < 0.05 vs. its corresponding control value; #*P* < 0.05 vs. its corresponding mock value

Since Kynu regulates tight junctions and tryptophan metabolism, we investigated whether this effect is mediated through downstream tryptophan metabolites. We evaluated the impact of XA, QA, and AA on barrier function by measuring the TEER in cholangiocyte monolayers under hypoxic conditions. Hypoxia significantly reduced TEER, while XA not QA or AA ameliorated this effect (Fig. 7A). Immunofluorescence analysis demonstrated that hypoxia disrupted zonula occludens-1 (ZO-1) protein localization at cell junctions, which was prevented by XA treatment (Fig. 7B). To assess the effect of XA on cholangiocyte responses to hypoxia, we quantified inflammatory cytokine levels, including IL-6, IL-10, TNF-α, and MCP-1 (Fig. 7C-F). Hypoxia significantly elevated IL-6 and MCP-1 secretion while suppressing IL-10 levels, and TNF-α remained unchanged. Under normoxia, XA had no effect on these cytokines. However, under hypoxia, XA reduced TNF-α, IL-6, and MCP-1 levels while upregulating IL-10. These findings suggest that XA modulates the inflammatory response to hypoxic stress in vitro.

The kynurenine pathway generates biologically active metabolite that interact with specific receptors, including aryl hydrocarbon receptor (with KA), N-methyl-D-aspartate receptor (with KA or QA), GluR2 (with XA). We therefore examined whether XA regulates GluR2/3 and CX50 expression in bile duct injury. Hypoxia downregulated GluR2 expression, which XA restored; CX50 levels remained unaffected by XA (Fig. 7G). Collectively, these data indicate that XA mitigates the hypoxia-induced increase in intercellular permeability, preserves tight junction integrity, and attenuates inflammatory responses in hypoxic environments.

### BSZ and XA protects against cMP-induced bile duct hyperplasia in long term

**Fig. 8:**
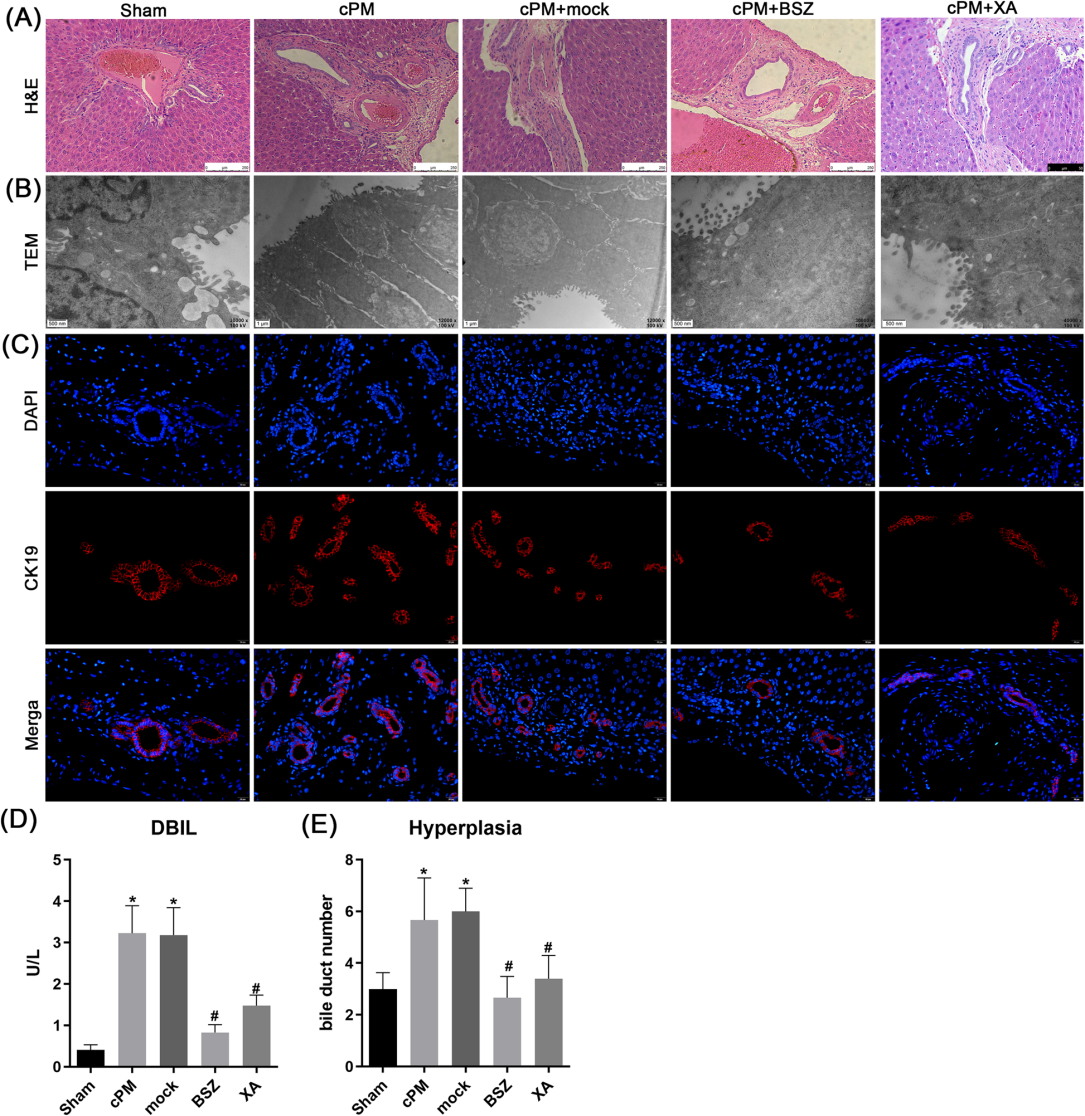
The effect of Kynu inhibition and XA on bile duct stricture and hyperplasia in long term. Intrahepatic bile duct histology. Shown is **A** H&E histology and **B** TEM from intrahepatic (portal tract × 400) (arrows indicate bile ducts). Note diminished portal periductal infammation and lack of intrahepatic biliary obstruction in the BSZ and XA group. **C** Intrahepatic bile duct hyperplasia. Shown is immunofluorescence stain for CK19 from intrahepatic (portal tract × 400) **D** Serum direct bilirubin. Shown is the mean ± SEM of serum direct bilirubin levels obtained at 7 d of life, demonstrating significant bilirubin reduction in BSZ and XA treated rats. **E** Hyperplasia was shown as bile duct number. *n* = 6 rats for each group. The results are depicted as the mean ± SD. Data from a representative (*n* = 3) experiment are shown. *P < 0.05 vs. sham group; # *P* < 0.05 vs. cPM group

From a therapeutic perspective, we hypothesized that modulating endogenous tryptophan metabolism through Kynu inhibition—thereby promoting xanthurenic acid (XA) production—might represent an attractive strategy. Consequently, we investigated the long-term effect of BSZ and XA administration on bile duct pathology. Mortality rates at one week were 10% (sham), 10% (cPM), 20% (mock), 10% (BSZ), and 10% (XA). Portal tracts with H&E staining revealed similar degrees of bile duct damage for five group. The hepatic cords of the rats were arranged in a regular and neat pattern, with no vacuolization or necrosis observed in the hepatocytes. However, compared to cPM and Mock group, BSZ and XA treated rats presented less mononuclear cell infiltration in the portal area, less bile duct hyperplasia and lack of luminal obstruction. These findings indicate that all five groups exhibited the restoration of hepatic structure, and BSZ and XA enhanced liver recovery and reduced bile duct hyperplasia (Fig. 8A). Serum direct bilirubin levels differed significantly among groups (*p* < 0.001). A significant increase in total bilirubin was observed on the 1 st week in cPM group compared to sham group (*p* < 0.001), which subsequently decreased following BSZ or XA treatment (Fig. 8D). Bile duct hyperplasia was further assessed via TEM and tissue staining with CK19. Compared to sham group, hyperplasia increased significantly in the cPM group by week 1 (Fig. 8B-C). In contrast, BSZ- or XA-treated rats exhibited significantly lower hyperplasia than both cPM and mock groups (Fig. 8E). These data demonstrate that BSZ or XA administration confers long-term protection against cPM-induced ischemic bile duct injury, as evidenced by improved bilirubin level, histological score and reduced hyperplasia.

## Discussion

The present study demonstrates that cPM-induced bile duct ischemic injury correlates with alterations in tryptophan metabolism. cPM upregulated Kynu expression, a key enzyme in Kynurenine pathway, leading to an imbalance in XA and QA synthesis. XA modulates ZO1 expression via GluR2/CX50, thereby improving impaired tight junction proteins and restoring biliary-barrier function. Inhibition of Kynu using shRNA or BSZ significantly protected against bile duct against ischemic injury, as evidenced by improved biochemical function, histological analysis, reduced bile duct necrosis/apoptosis, and decreased neutrophil infiltration. T This protective mechanism appears mediated through XA’s regulation of ZO1 expression via GluR2/CX50, maintaining biliary barrier integrity. Notably, long-term administration of the Kynu inhibitor BSZ ameliorated bile duct hyperplasia without causing mortality or hepatic abnormality.

Tryptophan, an essential amino acid requiring dietary supplemented, participates in protein biosynthesis and multiple metabolic processes (Modoux et al. 2021). Our transcriptome sequencing data showed that DEGs significantly enriched in cell tight junction pathways and metabolism pathways, such as Tryptophan metabolism, Pyruvate metabolism, Arginine and proline metabolism, Ascorbate and aldarate metabolism. Especially, a few Tryptophan metabolism-related DEGs were identified, including Kat1 and Kat3 between cHAFO and cPM, while Kynu and Haao were differentially expressed between iHAFO and iPM. These findings suggest that modulating either Kat or Kynu could replicate the bile duct-protective effects of tryptophan regulation. This hypothesis aligns with observed elevations in kynurenic acid (KA), XA, 3-hydroxyanthranilic acid (3-HAA), and QA levels. Previous studies support these observations. Y Mizoguchi et al. identified a hepatocyte metabolic pathway converting tryptophan to indole-3-acetic acid (IAA) via tryptamine in D-galactosamine-induced liver injury (Mizoguchi et al. 1988). Additionally, intraperitoneal administration of tryptophan (Ren et al. 2009) or D-Kynurenine (Kondo et al. 2009) significant increased KA levels in rodent liver. Our study provides the first evidence of tryptophan metabolism-related key enzymes from extrahepatic bile duct tissue. The extrahepatic kynurenine pathway contributed little to tryptophan degradation under normal conditions, but played a major role after immune activation (Badawy 2015). The pathway’s role in ischemic bile duct injury remains to be fully elucidated.

We investigated the effect of Kynu inhibition on bile duct ischemic injury in vitro and in vivo. The in vitro model employed cholangiocytes exposed to either normoxic or hypoxic conditions for 4 h, followed by normoxia for 1–4 h. Our findings demonstrated that Kynu inhibition under hypoxic conditions significantly reduced IL-6 and MCP-1 levels, but it showed no significant effect on IL-10 and TNF-α in hypoxia. These results suggest that Kynu may modulate cellular inflammatory responses in hypoxic environments in vitro.

Subsequently, we validated these findings using an experimental model of cPM rats subjected to either sham operations or 45 min of transient cPM occlusion followed by 24 h of hepatic reperfusion.

Multiple pathogenic factor may contribute to biliary complications after liver transplantation (Boeva et al. 2021), including ischemia-induced bile duct apoptosis, inflammation responses, blood-biliary barrier. Apoptosis represents a central mechanism in hepatic ischemia–reperfusion injury (Rüdiger et al. 2003). Inhibiting apoptosis, after liver insults, will inevitably improve the outcome of human liver and bile duct ischemic injury (Wang et al. 2021). Inflammatory cells infiltrations (neutrophils and monocytes/macrophages) and ductular proliferation contribute to ischemic biliary system injury (Zimmermann et al. 2012). Recent studies showed inflammatory cytokines such as TNF-α, IL-6, and MCP-1 contributed to the ischemic injury of bile duct (Malhi et al. 2006; Aller et al. 2019). In our cPM rat model, we confirmed the anti-inflammatory effects of Kynu inhibition, which attenuated bile ducts ischemic damage and demonstrated protective effects on cholangiocyte.

We hypothesized that ischemia inducs inflammatory factors, activates Kynu (secrete Tryptophan/Kynurenine, etc.), and exacerbates barrier damage either independently or synergistically. This process facilitates the translocation of kynurenic acid (KA) and quinolinic acid (QA) across the biliary barrier, contributing to hepatotoxic responses. Conversely, disruption of the blood-biliary barrier by tryptophan (Trp) or QA may facilitate pathogen entry, triggering subsequent inflammatory cascades. To investigate this mechanism, we analyzed alterations in Trp and KA metabolites. Following Trp conversion to kynurenine, kynurenine undergoes further metabolism: it is either converted to 3-HK by Kmo or to AA by Kynu. Additionally, Kynu catalyzes the conversion of 3-HK to 3-HAA, which ultimately metabolized to QA. The plasma levels of tryptophan and its metabolites, Kynurenine, 3-HK, HAA, KA, or the Tryptophan/Kynurenine ratio, have been linked to chronic and acute inflammation and implicated in ischemic injury (Zhang et al. 2020; Zheng et al. 2019). In our study, the bile duct ischemia increased levels of 3-HAA, AA, QA, or The Tryptophan/Kynurenine Ratio, underscoring Kynu’s pivotal role in bile duct ischemic damage. Inhibition of Kynu via shRNA or BSZ significantly reduced AA, 3-HAA, QA, and the Kyn/TRP Ratio, while increased XA level but had no significant effect on KA. As illustrated in Fig. 6, Kynu inhibition obstructs the Trp → 3-HAA → QA pathways are impeded, diverting metabolism toward Trp → KA or XA. This is consistent with our experimental findings. Previous studies suggest that XA or KA supplementation mitigates colitis severity by modulating intestinal epithelial cells (Michaudel et al. 2023). XA preserves mitochondrial function and attenuates myocardial ischemic injury (Lai et al. 2023). In contrast, QA, an agonist of the N-methyl-D-aspartate (NMDA) receptor, disrupts tight junction integrity in blood–brain barrier models (Št’astný et al. 2000). Furthermore, Kynu can directly hydrolyze kynurenine to form AA, which plays a significant role in myocardial, brain, and kidney injury. At the cellular level, we demonstrated that XA, but not QA or AA, modulates intercellular junctions and the expression of ZO1. Hypoxic cell models further confirmed that XA suppresses hypoxia-induced inflammatory cytokine upregulation. These findings implicate XA, a metabolite in the Kynu-mediated Try/Kyn pathway, may participate in PM-induced biliary ischemic injury via its regulation of intercellular junctions.

We then investigated the mechanism by which XA regulates ZO1 and tight junctions. ZO1, an intracellular component of tight and adherens junctions, also interacts with various connexins at gap junctions (Imafuku et al. 2023). A retinal study demonstrate that ZO1 forms distinct edges around Cx50 plaques, without co-localizing with connexin staining, suggesting its role in tight or adherens junctions around plaques rather than directly interacting with connexins. Concurrently, ZO1 forms intercellular junctions around CX50 at GluR2/3 (Puller et al. 2009). Furthermore, XA mimics mGlu2/3 receptor agonists by inhibiting cAMP generation in cortical neurons, with this inhibitory effect being significantly diminished in mGluR2 knockout mice (Fazio et al. 2015). In meninges, [3H]XA specifically binds to neuronal mGluR2/3 (Neale et al. 2013), and similar binding occurs in membrane of HEK-293 cells transfected with mGlu2 or mGlu3. These studies suggest that XA can activate GluR2/3. We also confirmed that XA upregulates GluR2/3 expression in the hypoxic bile duct cell model, which may be the mechanisms by which XA regulates ZO1.

In humans, Kats are encoded by four genes: Kat1, Kat2/Aadat, Kat3, and GOT2; while Kynu is encoded by a single gene. Our RNA-seq and subsequent verification data confirmed Kat2 and Kynu transcription in hepatic and extrahepatic bile duct tissues of both rats and Humans. The blood-biliary barrier, composed of tight junction between bile duct epithelial cells, maintains a 1000-fold higher bile salt concentration in bile canaliculi compared to portal blood, ensuring selective substance exchange (Maly and Landmann 2008). Increased barrier permeability may release XA, AA, KA, and QA from the bile duct to the liver, altering the ischemia-induced biliary damage. Indian ink permeability assays and ZO-1/CK19 staining revealed that shKynu and BSZ reduced ink leakage and ZO-1/CK19 expression. Occludin, claudins, and ZOs are the main components of tight junction (Furuse et al. 1993; Furuse et al. 1998). Damage to the tight junction between bile duct epithelial cells directly leads to impaired function of the blood-biliary barrier (Pradhan-Sundd et al. 2018; Santa Cruz et al. 2007). Notably, Kynu downregulation attenuated ischemia-induced barrier integrity loss.

Ischemic injury post-liver transplantation (LT) leads to nonanastomotic ischemic-type biliary lesions, including stricture and hyperplasia. Current liver ischemia–reperfusion treatments prioritize iPM over bile duct injury mitigation, despite risks of hyperplasia and infections. To address this gap, we evaluated a novel therapeutic strategy targeting XA production elevation and QA reduction via Kynu inhibition. The BSZ administration successfully modulated XA and QA level protecting rats from bile duct hyperplasia and injury within one week. This approach restores anti-inflammatory metabolites production while suppressing proinflammatory mediators like QA.

Despite the promising findings of this study, several limitations need to be considerated. First, the in vitro hypoxia model employed to investigate cholangiocyte injury may not fully replicate the complexity of ischemic injury in vivo. The hypoxia/reoxygenation duration may not precisely reflect the temporal dynamics of ischemia–reperfusion injury in the clinical setting. Second, although Kynu inhibition demonstrated significant protection against ischemic injury, the long-term effects of sustained Kynu inhibition and potential off-target effects were not thoroughly evaluated. Third, while the XA/GluR2/3/CX50-ZO1 axis was identified as a protective mechanism, its downstream signaling pathways and potential interactions with other metabolites (e.g., QA, KA, 3-HAA) or inflammatory cascades remain unclear. Finally, as the findings were primarily derived from rat models, their translational relevance to human physiology remains uncertain, necessitating further validation in clinical settings.

## Conclusion

In summary, our study shows a novel relationship between Trp metabolism and bile duct injury during liver IRI. Our data demonstrate that down-regulation of Kynu expression preserve bile duct function and maintains the integrity of the blood-biliary barrier. The protective mechanism appears to be mediated through Kynu inhibition-induced elevation of XA levels, which up-regulate GluR2/3/CX50-ZO1 axis and improve impaired tight junction. Together, this study paves the way for new therapeutic strategies targeting Kynu inhibition to mitigate bile duct ischemic injury.

## Supplementary Information

Supplementary Material 1.

Supplementary Material 2.

## Data Availability

No datasets were generated or analysed during the current study.

## Abbreviations

KA: Kynurenic acid
3-HK: 3-Hydroxykynurenine
3-HAA: 3-Hydroxyanthranilic acid
AA: Anthranilic acid
QA: Quinolinic acid
Kynu: Kynureninase
Haao: 3-hydroxyanthranilic acid dioxygenase
Kat: Kynurenine aminotransferase
Kmo: Kynurenine 3-monooxygenase
NAD: Nicotinamide adenine dinucleotide
IL: Interleukin
cPM: Continuous Pringle maneuver
