# Supplementary material for: Kynu inhibition mitigates bile duct ischemic injury by rewiring tryptophan metabolism to restore tight junction integrity
Source: Mol Med. 2025 Aug 19;31:279. doi: 10.1186/s10020-025-01310-6 (PMC12366207; doi:10.1186/s10020-025-01310-6)
Supplement: Supplementary file 1 — Supplementary Material 1. [file 10020_2025_1310_MOESM1_ESM.docx]

Table S1 The sequence of primers for qPCR in this study.

| Genes | Sequences |
| --- | --- |
| Claudin-1 | GTCTTTGACTCCTTGCTGAATCTG |
|  | CACCTCATCGTCTTCCAAGCAC |
| Claudin-3 | GCCTTCATCGGCAGCAACATCA |
|  | AGCGAGTCGTACACCTTGCACT |
| ZO-1 | GTCCAGAATCTCGGAAAAGTGCC |
|  | CTTTCAGCGCACCATACCAACC |
| Kmo | GCACTGAATGCCTGCTTTCTTGC |
|  | CCAGACCAATGGCTTTCAAGGC |
| Kynu | GTATGCGGATGGTAAAGCCACG |
|  | CACTGAACAGGATCACGGCGAT |
| Kat2 | CTGCCTTCACTGTGGAAAACGG |
|  | GTTTTAGCCAGGACAGAAGTTCTG |
| Haao | CCTGAGACAGAATGTGGACGTG |
|  | CTTGTGTTCGCTCCCAGGCATA |

Table S2 Key resources in this study.

| Genes | Sequences |
| --- | --- |
| Claudin-1 | Proteintech (28674-1-AP) |
| Claudin-3 | Proteintech (16456-1-AP) |
| Claudin-5 | Proteintech (29767-1-AP) |
| Occludin | Proteintech (27260-1-AP) |
| Ki67 | Proteintech (27309-1-AP) |
| MPO | Proteintech (22225-1-AP) |
| CK19 | Proteintech (14965-1-AP) |
| ZO-1 | Proteintech (21773-1-AP) |
| Kmo | Proteintech (10698-1-AP) |
| Kynu | Proteintech (11796-1-AP) |
| Kat2 | Proteintech (13031-1-AP) |
| Haao | Proteintech (12791-1-AP) |
| β-actin | Proteintech (66009-1-Ig) |

Table S3 DEGs between CPM group and CHAFO group (log2Fold>1.5, q<0.05)

| Fold-change of CHAFO/CPM | | | | | |
| --- | --- | --- | --- | --- | --- |
| Gene | Down-regulate | Q value | Gene | Up-regulate | Q value |
| LOC108348266 | -5.704 | 3.64E-05 | RGD1309489 | 5.4536 | 3.20E-03 |
| LOC103691594 | -5.326 | 5.36E-03 | LOC108352932 | 3.382772 | 1.84E-05 |
| Kynu | -4.807 | 8.08E-10 | Cldn18 | 3.306814 | 2.36E-03 |
| Cyp3a9 | -3.348 | 5.06E-05 | Chgb | 3.10334 | 9.37E-12 |
| Egr2 | -3.100 | 2.75E-13 | LOC108353528 | 3.058719 | 3.09E-04 |
| Orm1 | -2.534 | 2.76E-02 | Atg9b | 2.620791 | 4.02E-09 |
| Aldh1b1 | -2.410 | 1.51E-02 | LOC498063 | 2.463744 | 4.07E-02 |
| Areg | -2.256 | 2.90E-02 | Fstl4 | 2.405133 | 2.04E-02 |
| Lcn2 | -2.110 | 3.56E-04 | Cela1 | 2.265351 | 1.41E-02 |
| Cyp2d2 | -2.095 | 1.93E-02 | Slc26a9_1 | 2.197925 | 1.50E-04 |
| Serpina6 | -2.089 | 1.69E-07 | Sectm1b | 2.042288 | 2.56E-08 |
| LOC108348093 | -1.938 | 9.31E-03 | Ctrb1 | 1.996895 | 1.42E-02 |
| Ugt1a1 | -1.890 | 3.78E-02 | Pnlip | 1.964514 | 4.83E-02 |
| Slc17a4 | -1.852 | 4.63E-02 | Pdia2 | 1.92859 | 1.99E-02 |
| Aass | -1.818 | 5.13E-03 | Ctrl | 1.913796 | 3.79E-02 |
| A2m | -1.792 | 2.66E-03 | Arhgdig | 1.872172 | 1.05E-02 |
| Fmo2 | -1.768 | 7.32E-03 | Cela3b | 1.84492 | 1.77E-02 |
| Kyat3 | -1.662 | 7.30E-04 | Ctrc | 1.818411 | 3.65E-02 |
| Rnf125 | -1.648 | 1.48E-03 | LOC100911864 | 1.784406 | 2.21E-06 |
| Gpld1 | -1.633 | 8.19E-03 | Nccrp1 | 1.679811 | 3.64E-02 |
| Sugct | -1.594 | 1.88E-03 | Pnliprp2 | 1.670239 | 1.92E-02 |
| Ces2j | -1.526 | 6.35E-03 | Reg3a | 1.656221 | 9.41E-03 |
|  |  |  | Zg16 | 1.650514 | 3.76E-02 |
|  |  |  | Cpa1 | 1.644307 | 3.56E-02 |
|  |  |  | Tmed6 | 1.630888 | 3.98E-02 |
|  |  |  | Shisa4 | 1.577941 | 9.03E-03 |
|  |  |  | Clu | 1.534212 | 6.21E-03 |
|  |  |  | Reg3b | 1.505142 | 1.42E-02 |

Table S4 GO terms and KEGG pathway between CPM and CHAFO after comparing with sham group.

| ID | Description | GeneRatio | pvalue |
| --- | --- | --- | --- |
| GO terms |  |  |  |
| GO:0032496 | response to lipopolysaccharide | 40/1140 | 2.01E-08 |
| GO:0001889 | liver development | 28/1140 | 2.01E-08 |
| GO:0051591 | response to cAMP | 21/1140 | 1.17E-07 |
| GO:0043434 | response to peptide hormone | 26/1140 | 9.37E-07 |
| GO:0006953 | acute-phase response | 14/1140 | 6.98E-06 |
| GO:0051384 | response to glucocorticoid | 23/1140 | 9.81E-06 |
| GO:0007568 | aging | 43/1140 | 1.25E-05 |
| GO:0014070 | response to organic cyclic compound | 33/1140 | 1.25E-05 |
| GO:0007584 | response to nutrient | 22/1140 | 0.000108 |
| GO:0042493 | response to drug | 50/1140 | 0.000254 |
| GO:0032869 | cellular response to insulin stimulus | 21/1140 | 0.000383 |
| GO:0033993 | response to lipid | 10/1140 | 0.001222 |
| GO:0032570 | response to progesterone | 12/1140 | 0.00211 |
| GO:0020037 | heme binding | 24/1140 | 0.003748 |
| GO:0004497 | monooxygenase activity | 10/1140 | 0.005988 |
| GO:0010043 | response to zinc ion | 10/1140 | 0.005988 |
| GO:0005506 | iron ion binding | 25/1140 | 0.005988 |
| GO:0006805 | xenobiotic metabolic process | 13/1140 | 0.009789 |
| GO:0004029 | aldehyde dehydrogenase (NAD) activity | 5/1140 | 0.017593 |
| GO:0045471 | response to ethanol | 21/1140 | 0.017593 |
| GO:0001666 | response to hypoxia | 24/1140 | 0.018602 |
| GO:0010033 | response to organic substance | 16/1140 | 0.018602 |
| GO:0009612 | response to mechanical stimulus | 13/1140 | 0.018602 |
| GO:0043627 | response to estrogen | 13/1140 | 0.020625 |
| GO:0030170 | pyridoxal phosphate binding | 9/1140 | 0.038257 |
| GO:0071222 | cellular response to lipopolysaccharide | 20/1140 | 0.04688 |
| KEGG pathway |  |  |  |
| ko05150 | Staphylococcus aureus infection | 17/529 | 1.68E-08 |
| ko00380 | Tryptophan metabolism | 14/529 | 2.58E-06 |
| ko00620 | Pyruvate metabolism | 12/529 | 3.70E-05 |
| ko00330 | Arginine and proline metabolism | 12/529 | 9.01E-05 |
| ko05204 | Chemical carcinogenesis | 17/529 | 0.000103 |
| ko00040 | Pentose and glucuronate interconversions | 10/529 | 0.000153 |
| ko00260 | Glycine, serine and threonine metabolism | 10/529 | 0.000242 |
| ko00140 | Steroid hormone biosynthesis | 15/529 | 0.000332 |
| ko00640 | Propanoate metabolism | 9/529 | 0.000394 |
| ko00053 | Ascorbate and aldarate metabolism | 8/529 | 0.000496 |
| ko00340 | Histidine metabolism | 7/529 | 0.000775 |
| ko00982 | Drug metabolism - cytochrome P450 | 13/529 | 0.000928 |
| ko04972 | Pancreatic secretion | 15/529 | 0.002478 |
| ko00280 | Valine, leucine and isoleucine degradation | 10/529 | 0.00498 |
| ko00830 | Retinol metabolism | 13/529 | 0.006437 |
| ko04060 | Cytokine-cytokine receptor interaction | 26/529 | 0.009413 |
| ko04974 | Protein digestion and absorption | 12/529 | 0.013496 |
| ko00980 | Metabolism of xenobiotics by cytochrome P450 | 10/529 | 0.016729 |
| ko00591 | Linoleic acid metabolism | 7/529 | 0.026655 |
| ko05140 | Leishmaniasis | 9/529 | 0.03227 |
| ko00500 | Starch and sucrose metabolism | 8/529 | 0.033151 |
| ko00627 | Aminobenzoate degradation | 3/529 | 0.042616 |
