# Supplementary figures and images for: Kynu inhibition mitigates bile duct ischemic injury by rewiring tryptophan metabolism to restore tight junction integrity
Source: Mol Med. 2025 Aug 19;31:279. doi: 10.1186/s10020-025-01310-6 (PMC12366207; doi:10.1186/s10020-025-01310-6)

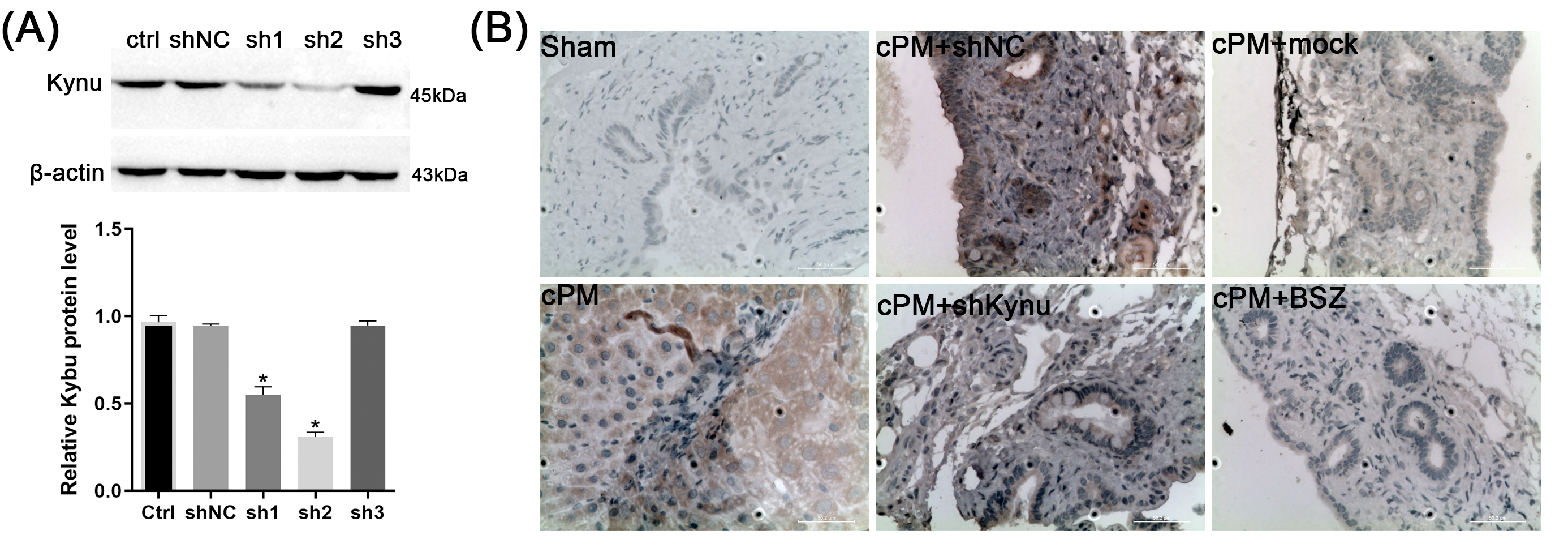

Supplement: Supplementary file 2 — Supplementary Material 2. [file 10020_2025_1310_MOESM2_ESM.tif]
